# Warm Autoimmune Hemolytic Anemia and Pure Red Cell Aplasia during a Severe COVID-19 B.1.1.7 Infection

**DOI:** 10.3390/idr14030044

**Published:** 2022-06-02

**Authors:** Anukul Ghimire, Jaye Platnich, Utkarsh Chauhan

**Affiliations:** 1 Department of Medicine, University of Alberta, Edmonton, AB T6G 2G3, Canada; platnich@ualberta.ca; 2 Faculty of Medicine and Dentistry, University of Alberta, Edmonton, AB T6G 2R7, Canada; uchauhan@ualberta.ca

**Keywords:** warm autoimmune hemolytic anemia, pure red cell aplasia, COVID-19, parvovirus B19

## Abstract

Warm autoimmune hemolytic anemia (AIHA) is a rare complication of COVID-19 infection. We report a case of warm AIHA in a patient with COVID-19 pneumonia treated with methylprednisolone and several red blood cell transfusions. Despite treatment of the warm AIHA, the patient’s reticulocyte count remained low, and his biochemical markers were suggestive of pure red cell aplasia, which was later attributed to a concurrent parvovirus B19 infection. This case highlights an unusual situation of two separate hematological processes caused by two separate and simultaneous viral infections.

## 1. Case Report

Viral infections are well known triggers of hematologic and rheumatologic disease. Since the start of the pandemic, SARS-CoV-2 infection has been associated with numerous systemic effects, including autoimmune and hematologic manifestations [1,2]. In this report, we describe a case of COVID-19 induced autoimmune hemolytic anemia (AIHA) occurring concurrently with a parvovirus B19 mediated pure red cell aplasia.

A 29-year-old male, who tested positive for the B.1.1.7 variant of COVID-19 ten days prior, presented to the Emergency Department (ED) after a syncopal episode at home. He had experienced 2 weeks of subjective fevers, rhinorrhea, anorexia, malaise, and 2–3 days of dyspnea. His comorbidities included obesity, mild asthma, and gastroesophageal reflux. His only home medication was pantoprazole 40 mg daily.

On examination, he was afebrile, with a blood pressure of 118/61 mmHg, a heart rate of 124 beats per minute, and a respiratory rate of 43 breaths/minute. He was saturating 92–95% on 4 L of supplemental oxygen. He was markedly pale, had significant scleral icterus, and was diffusely wheezy on respiratory exam. His Foley catheter was draining rusty brown urine.

His last available bloodwork prior to presentation was over two years ago and was unremarkable. On presentation, he was found to have significant anemia, leukocytosis, thrombocytosis, and an acute kidney injury (Table 1). A hemolytic workup revealed a positive direct antiglobulin test (IgG and C3), an undetectably low haptoglobin, elevated lactate dehydrogenase (LDH), elevated bilirubin, and elevated plasma free hemoglobin. His reticulocyte count was inappropriately low-normal. His urinalysis showed 300 erythrocytes/uL, but no red blood cells on microscopy. A chest radiograph revealed patchy opacities in both lungs consistent with COVID-19 pneumonia.

Within 4 h of presentation, he became progressively hypoxemic and required urgent intubation and admission to the Intensive Care Unit (ICU). He was started on methylprednisolone 500 mg intravenously daily for treatment of warm AIHA and was transfused 6 units of matched blood. His formal peripheral blood smear was reported later that day and demonstrated spherocytes, consistent with immune-mediated hemolytic anemia and auto-agglutination consistent with possible cold reacting auto-antibody; however, his cold agglutinin titre was negative (Table 1). A formal diagnosis of warm AIHA with reticulocytopenia, possibly in the setting of concurrent pure red cell aplasia, (PRCA) was made.

**Figure 1:**
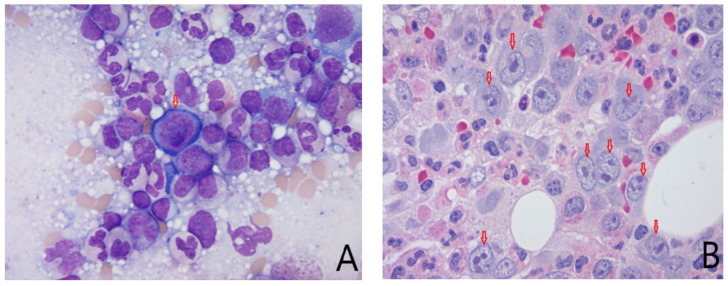
Images from the patient’s bone marrow biopsy samples: (**A**) Bone marrow aspirate. Arrow shows a proerythroblast with possible viral inclusion (difficult to distinguish from unusually large nucleoli). (**B**) Bone marrow trephine: Erythroid cells (arrows) are decreased in number and severely left-shifted with nearly complete arrest at the pro-erythroblast stage.

His ICU course was short: he was extubated on post-admission day 2 and was transferred to the ward the same day. A workup for secondary infectious, autoimmune, and malignant etiologies (Table 2) for warm AIHA was negative. He continued to demonstrate reticulocytopenia despite his ongoing anemia, and a work-up for pure red cell aplasia yielded a positive parvovirus B19 IgM but a negative parvovirus B19 nucleic acid titre (NAT). He underwent a bone marrow biopsy that showed decreased erythroid cells and a severe left shift, with near-complete arrest at the pro-erythroblast stage. Furthermore, a number of proerythroblasts with suspected viral inclusions were observed, although it was not possible to discern these from large nuclei (Figure 1).

**Figure 2:**
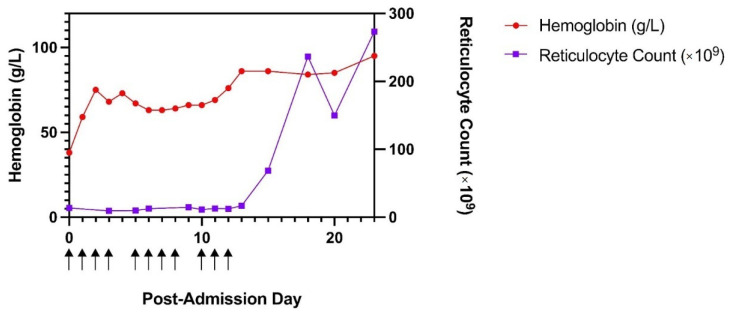
A graph of the patient’s hemoglobin and reticulocyte count over time: The patient’s hemoglobin (g/L) and reticulocyte count (×10^9^) charted over time, as defined by days post-admission to hospital on 10 April 2021. Arrows under the *x*-axis indicate days on which the patient received one or more blood transfusions.

It was felt that the warm AIHA was due to COVID-19; however, a transient parvovirus infection likely caused PRCA, resulting in transfusion-dependency despite appropriate treatment of his AIHA. He was stepped down to dexamethasone for treatment of his warm AIHA in the setting of his active COVID-19 infection. His reticulocyte count recovered spontaneously approximately 2 weeks after admission (Figure 2).

## 2. Discussion

We report a case of a patient who had evidence of warm AIHA likely due to a B.1.1.7 variant of COVID-19. COVID-19-associated AIHA has been previously described [1,2], Furthermore, this patient had evidence of recent parvovirus B19-mediated pure red cell aplasia, which significantly worsened his anemia in the setting of ongoing hemolysis. Co-infection with SARS-CoV-2 and parvovirus B19 has been found to cause severe cardiac, neurologic, and hematologic manifestations [3,4,5,6,7].

COVID-19 has been linked to several autoimmune conditions including autoimmune cytopenias, cutaneous vasculitis, encephalitis, and Guillain Barre syndrome [1]. Among hematologic autoimmune disorders, immune thrombocytopenic purpura (ITP) and AIHA are the most common [2]. Molecular mimicry has been proposed as a possible mechanism underlying these autoimmune phenomena given significant sequence homology between the COVID-19 virus spike protein and human proteins [8,9]. Ankyrin-1 is an erythrocyte membrane protein that shares an immunogenic epitope with spike protein; this may promote immunological cross reactivity and lead to AIHA [10]. AIHA typically is seen 9 +/− 5 days after the onset of COVID-19 infection presentation [2].

AIHA is an uncommon entity with estimated prevalence of 17 per 100,000 and incidence of 1 to 3 per 100,000/year [11]. Most cases pertain to the “warm” subtype [12]. The various etiologies of secondary AIHA are enumerated in Table 2. The precise link between COVID-19 and AIHA has yet to be elucidated. Taherifard et al. reviewed 58 studies looking at hematological autoimmune disorders induced from COVID-19 and found 22 cases of AIHA up until 19 December 2020 [2]. Most of the COVID-19-associated AIHA cases have been attributed to acquired cold agglutinin syndrome and, to our knowledge, all have been attributed to wild-type COVID-19 [2,13].

The symptoms of warm AIHA mirror those associated with anemia in general: fatigue, subjective weakness, pallor, pre-syncope/syncope and, in severe cases, shortness of breath. Symptoms more specific to hemolysis include jaundice and dark urine [12,14]. The most common physical exam findings include pallor and jaundice.

Laboratory investigations in warm AIHA demonstrate positive hemolytic markers including high LDH, high unconjugated bilirubin, high plasma free hemoglobin, high reticulocyte count, low haptoglobin, and the presence of hemoglobinuria [12,14]. A peripheral blood smear will often reveal the presence of spherocytes and polychromasia. The most useful diagnostic test in this setting is the direct antiglobulin test (DAT), which can distinguish between immune-mediated and non-immune-mediated hemolytic anemias [12,14]. IgG-positive DATs are generally suggestive of warm AIHA, whereas C3-positive DATs are more consistent with cold AIHA. It is not uncommon for people to have both IgG-positive and C3-positive DATs in warm AIHA. An IgG-positive DAT in the presence of positive hemolytic markers and the right clinical presentation is diagnostic of warm AIHA.

The mainstay of initial treatment of warm AIHA is supportive transfusions with matched packed RBCs and high-dose steroids such as a methylprednisolone pulse and/or prednisone 1 mg/kg daily for a prolonged course and taper [12,14]. Approximately 70–90% of patients respond to steroids alone without the need for additional agents. Rituximab and mycophenolate mofetil are some of the second line agents in steroid non-responders. Intravenous immune globulin has limited efficacy as a single agent in AIHA. The second key intervention is to treat the underlying cause of the AIHA: in the present case, the patient’s ongoing B.1.1.7. variant COVID-19 infection. Other standards of care in AIHA patients include folic acid supplementation and deep vein thrombosis (DVT) prophylaxis given the profound folate deficiency and prothrombotic effects associated with hemolysis respectively [14].

The present case was complicated by the concurrent presence of PRCA with evidence of recent parvovirus B19 infection, suggested by parvovirus IgM antibodies and suspected viral inclusions in bone marrow cells. PRCA is a state characterized by the cessation of RBC production without associated reductions in other cell lines. This is most often seen with neoplasms of lymphoid cells (including plasma cells), but may also occur secondary to medications, autoimmune conditions, and infections such as parvovirus B19 [15]. This case also highlights some important differentials for hemolysis without reticulocytosis, including primary bone marrow disorders, drug or alcohol-induced myelosuppression, micronutrient deficiency, and autoantibody effect on erythrocyte progenitors in the bone marrow.

During states of RBC destruction, parvovirus B19 is known to cause temporary arrest of erythropoiesis in bone marrow [15]. In our case, this was demonstrated by the patient’s inability to increase reticulocyte production despite significant ongoing hemolysis. A previous experimental study showed that interruption of erythropoiesis occurred in the second week after inoculation and at the end of the viremic phase [15]. Thus, the negative parvovirus DNA titres measured in our patient’s serum may suggest that the patient had already passed the viremic phase and was having significant red cell aplasia that was unmasked by his COVID-19-induced hemolysis.

One limitation of this report is that we were not able to perform viral quantification on the bone marrow samples; we only have indirect evidence of parvovirus infection including the IgM titer and possible viral inclusions in rare proerythroblasts on bone marrow biopsy. In a case complicated by evidence of two separate viral infections and two distinct hematologic processes, bone marrow aspirate viral quantification may have confirmed the etiology of the PRCA.

It is possible that COVID-19 may have been partially or completely responsible for the disruption in erythropoiesis. However, this has not been previously described in the literature. Furthermore, the timing of his bone marrow recovery is very typical of parvovirus B19-associated PRCA [15]. Therefore, in accordance with Occam’s razor, his PRCA was attributed to a transient, but remarkably timed, parvovirus B19 infection superimposed on an active COVID-19 infection.

## 3. Conclusions

Variant strains of COVID-19 can induce autoimmune hemolytic anemia, which should be suspected in any patient with severe anemia and abnormal hemolytic markers. In our clinical scenario, a patient presented with warm AIHA secondary to B.1.1.7 COVID-19. The patient had prolonged transfusion-dependency despite appropriate treatment with steroids. Reticulocytopenia during hemolysis should prompt investigations to identify a cause of bone marrow suppression; parvovirus B19 can exacerbate anemia through pure red call aplasia.

## Figures and Tables

**Table 1:** Summary of select patient laboratory investigations on presentation.

Investigation (Units)	Result (Normal Range)
**Hematology**	
Hemoglobin (g/L)	38 (135–175)
Mean corpuscular volume (MCV) (fL)	98 (80–100)
Mean corpuscular hemoglobin concentration (MCHC) (g/L)	322 (310–360)
Leukocytes (×10^9^/L)	15.8 (4.0–11.0)
Platelets (×10^9^/L)	549 (140–400)
**General Chemistry**	
Sodium (mmol/L)	136 (135–145)
Potassium (mmol/L)	3.4 (3.5–5.0)
Chloride (mmol/L)	98 (98–112)
Bicarbonate (mmol/L)	21 (20–32)
Anion gap (mmol/L)	17 (5–10)
Creatinine (μmol/L)	133 (50–120)
eGFR (mL/min/1.73 m^2^)	62 (>59)
Urea (mmol/L)	11.2 (3.0–8.0)
Osmolality (mmol/kg)	304 (280–300)
Alanine aminotransferase (ALT) (U/L)	133 (<60)
Aspartate aminotransferase (AST) (U/L)	85 (<40)
Alkaline phosphatase (ALP) (U/L)	58 (40–120)
Lipase (U/L)	143 (0–60)
Albumin (g/L)	33 (30–45)
Calcium (mmol/L)	2.09 (2.10–2.60)
Magnesium (mmol/L)	0.91 (0.70–1.00)
Glucose, random (mmol/L)	11.2 (3.3–11.0)
C-reactive protein (CRP) (mg/L)	117.9 (<8.0)
Thyroid stimulating hormone (TSH) (mU/L)	2.14 (0.20–4.00)
Iron (μmol/L)	45 (8–35)
Total iron binding capacity (TIBC) (μmol/L)	58 (40–75)
Iron saturation index	0.78 (0.12–0.60)
Ferritin (μg/L)	2847 (30–500)
Vitamin B12 (pmol/L)	378 (>110)
Folate (nmol/L)	21.2 (>/=10)
**Coagulation**	
International normalized ratio (INR)	1.3 (0.8–1.2)
Partial thromboplastin time (PTT) (s)	25 (24–39)
Fibrinogen (g/L)	7.9 (2.0–4.0)
**Hemolytic Workup**	
Total bilirubin (μmol/L)	84 (<20)
Haptoglobin (g/L)	<0.10 (0.30–2.00)
Lactate dehydrogenase (LDH) (U/L)	1093 (100–225)
Plasma hemoglobin (mg/L)	57 (<50)
Reticulocyte absolute count (×10^9^/L)	13.5 (20–120)
Reticulocyte percentage (%)	1.2 (0.4–2.0)
Direct antiglobulin test (DAT)	IgG and C3 Positive (Negative)
Cold agglutinin titre	Negative (Negative)
Peripheral blood smear	Critical anemia with spherocytes and auto agglutination consistent with immune mediated hemolytic anemia with possible cold reacting autoantibody
**Urinalysis-Macroscopic**	
Specific Gravity	1.020 (1.005–1.030)
pH	5.0 (5.0–8.0)
Protein (g/L)	≥3.0 (Negative)
Blood (Ery/μL)	300 (Negative)
Urine was negative for glucose, leukocytes, nitrites, and ketones	
**Urinalysis-Microscopic**	
WBC (/HPF)	11–20 (0–5)
RBC (/HPF)	0–2 (0–2)
Bacteria (/HPF)	0–20 (0–20)

**Table 2:** Overview of the secondary causes of warm autoimmune hemolytic anemia.

Autoimmune	Inflammatory bowel disease (IBD)
	Rheumatoid arthritis
	Systemic sclerosis
	Systemic lupus erythematosus (SLE)
Drugs	Antibiotics
	Anti-malarials
	Anti-cancer agents
	Non-steroidal anti-inflammatory drugs (NSAIDS)
	Others
Immunodeficiencies	Inherited immunodeficiencies (e.g., combined variable immunodeficiency (CVID))
	Hypogammaglobulinemia
	Post-hematologic stem cell transplant
	Post-solid organ transplant
Infections	Epstein Barr virus (EBV)
	Hepatitis C virus (HCV)
	Hepatitis E virus (HEV)
	Human immunodeficiency virus (HIV)
Malignancies/Lymphoproliferative Disorders	Chronic lymphocytic leukemia (CLL)
	Hodgkin/non-Hodgkin lymphoma
	Plasma cell dyscrasias
	Solid tumors
Other	Babesiosis
	Brown recluse spider bite
	Pregnancy
	Idiopathic

## Data Availability

All available data is included in the manuscript.
